# Experiences of racism among Aboriginal and Torres Strait Islander adults living in the Australian state of Victoria: a cross-sectional population-based study

**DOI:** 10.1186/s12889-019-6614-7

**Published:** 2019-03-14

**Authors:** Alison Markwick, Zahid Ansari, Darren Clinch, John McNeil

**Affiliations:** 1 Department of Epidemiology and Preventive Medicine, School of Public Health and Preventive Medicine, Monash University, 553 St Kilda Rd, Melbourne, Victoria 3004 Australia; 2 Aboriginal Health and Wellbeing Branch, Department of Health and Human Services, State Government of Victoria, 50 Lonsdale Street, Melbourne, Victoria 3000 Australia

**Keywords:** Experiences of racism, Indigenous status, Social determinants, Social capital, Socioeconomic status, Education, Lifestyle risk factors, Australia, Victoria

## Abstract

**Background:**

Racism is a key determinant of the health of Indigenous Australians that may explain the unremitting gap in health and socioeconomic outcomes between Indigenous and non-Indigenous Australians. We quantified the population-based prevalence of experiences of racism of Indigenous adults in the Australian state of Victoria and investigated whether this was independent of social determinants and lifestyle risk factors.

**Methods:**

We combined data from the 2011, 2012 and 2014 Victorian Population Health Surveys to obtain a sample size of 33,833 Victorian adults, including 387 Indigenous adults. The survey is a cross-sectional, population-based, computer-assisted telephone interview survey conducted annually. Using logistic regression, experiences of racism was the dependent variable and Indigenous status the primary independent variable of interest. Secondary independent variables included age, sex, rurality, socioeconomic status, social capital, and lifestyle risk factors.

**Results:**

Indigenous Victorian adults were four times more likely than their non-Indigenous counterparts to have experienced racism in the preceding 12 months; odds ratio (OR) = 4.3 (95% confidence interval (CI): 3.2–5.8). Controlling for social determinants and lifestyle risk factors attenuated, but did not eliminate, the association between experiences of racism and Indigenous status; OR = 3.1 (95% CI: 2.2–4.3). The social determinants of age and social trust made the largest contribution to the attenuation of the association. Education also had a large impact on the association, but in the opposite direction, suggesting that a low level of education may be protective against experiences of racism. When the non-Indigenous comparison group consisted of adults of mainly Anglo-Celtic origin, Indigenous adults were seven times more likely (OR = 7.2; 5.3–9.7) to have experienced racism.

**Conclusions:**

Racism directed against Indigenous Victorians is significant and cannot be ascribed to any specific attributes such as socioeconomic status or lifestyle risk factors. We argue that a human rights-based approach to policy-making for the elimination of systemic and interpersonal racism offers an opportunity and viable alternative to current policy-making, that continues to be dominated by a paternalistic approach that reinforces racism and the resulting inequities.

**Please note:**

Throughout this document, the term Indigenous is taken to include people of Aboriginal and Torres Strait Islander descent. While not our preferred term, Indigenous is used in preference to Aboriginal and Koori as not all Indigenous people living in Victoria are Aboriginal or Koori. We recognise that, with the exception of the term ‘Koori’, these terms are Eurocentric having been imposed upon a people of many nations with distinct languages and cultures. The use of such terms is akin to referring to the peoples of the continent of Europe as ‘Europeans’.

## Background

The history of Indigenous people in Australia over the last two hundred years since the arrival of Europeans is one of great suffering. Genocide, the introduction of European diseases, dispossession, subjugation and segregation reduced the Indigenous population by 90% between 1788 and 1900 [1]. A conservative estimate indicates that prior to European contact there were approximately 15,000 Indigenous Australians living in the state of Victoria; that number was reduced to approximately 850 by 1901 [2].

Today, Indigenous Australians continue to face interpersonal and institutional racism which creates and sustains their lower socioeconomic status by excluding them from economic opportunities and land ownership. Moreover, Indigenous men and women can expect to live 10.6 and 9.5 years less than non-Indigenous men and women respectively [3]. A large and growing body of evidence consistently implicates racism as a key determinant of the health of Indigenous Australians [4, 5].

However, one of the most persistent aspects of today’s discourse regarding racism in Australia is the very denial of its existence [6]. A review of the linguistic and discursive patterns of contemporary speech in both informal and formal (parliamentary debates, political speeches, and the media) settings in Australia concluded that the social taboo against openly expressing racist beliefs has led to the development of strategies that present negative views of minority groups as reasonable and justified, while exonerating the speaker from charges of racism. This serves to constrain political efforts to address racism thus reinforcing racism [6].

In this paper we sought to: (a) quantify the population-based prevalence of racism experienced by Indigenous Australians who lived in the state of Victoria; (b) determine if experiences of racism among Indigenous Victorians are independent of lifestyle risk factors and social determinants, such as socioeconomic status, that are often used to justify negative stereotypes; (c) describe potential pathways by which experiences of racism impacts on Indigenous Victorians to create inequalities in health; and (d) identify key points of intervention and potential strategies to combat racism.

On an individual level, racism refers to the beliefs and attitudes that members of certain groups have of their superiority in relation to other groups who are regarded as inferior, based on race, ethnicity or cultural background [7]. Those who are assumed to be inferior are treated differently and unfavourably.

At a societal level, racism can be defined as “… that which maintains or exacerbates inequality of opportunity among ethnoracial groups” and racial discrimination as the racist behaviours and practices that result in inequality of opportunity among ethnoracial groups [8].

## Methods

### Data source

The Victorian Population Health Survey is an annual cross-sectional population-based survey that collects information on the health of adults who live in the Australian state of Victoria [9]. Random digit dialling of landline telephone was used to randomly select adults aged 18 years or older who lived in private dwellings.

The views expressed in this article are those of the authors and do not necessarily represent those of the Victorian Department of Health and Human Services or the Victorian Government of Australia.

### Sample size

We merged three Victorian Population Health Survey datasets to attain a sufficient number of adults who identified as Indigenous. This included data from the 2011 survey (33,673 participants), the 2012 survey (7533 participants), and the 2014 survey (33,654 participants). The sample size of the 2011 and 2014 surveys was based on recruiting approximately 426 participants for each of the 79 local government areas of Victoria, while the 2012 survey was based on recruiting approximately 900 participants for each of the 8 departmental regions. The sample size was based on detecting a variable of interest with a prevalence of 7.5%, confidence interval of 5.0 to 10.0%, and a relative standard error of 17%.

We deleted all non-Indigenous participants from the 2011 and 2012 surveys because they were not asked about experiences of racism. The combined dataset was stratified by departmental region and the final sample size was 33,833, which included 387 Indigenous participants.

### Response rate

The response rate was defined as the proportion of households where contact was made and an interview completed. It was 67% for the 2011 survey, 69% for the 2012 survey, and 70% for the 2014 survey.

### Weighting

To reduce participation bias, we reweighted the survey data to reflect the age/sex/geographic distribution of the census Indigenous and non-Indigenous resident population of Victoria in 2011 and the probability of selection of the household and the participant within the household. We then normalised the resulting weights to add up to the sample total in order to maximise the accuracy of the standard errors [10].

### Ethics statement

The survey was approved by the Victorian Department of Health Human Research Ethics Committee in accordance with the guidelines of the Declaration of Helsinki. Since the Victorian Population Health Survey is a general population health survey, there was no consideration of the Guidelines for Ethical Research in Australian Indigenous Studies.

### Variables

Experiences of racism were assessed by asking the following question in the 2011 and 2012 survey: “How often, if at all, have you received unfair treatment in the last 12 months because you are an Aboriginal or Torres Strait Islander?” Since only Indigenous participants were asked about experiences of racism in the 2011 and 2012 surveys, all non-Indigenous participants of these two surveys were eliminated from the combined dataset. In the 2014 survey all participants, regardless of Indigenous status, were asked the question “In the last 12 months, have you experienced discrimination or been treated unfairly because of your racial, ethnic, cultural, or religious background?” Although not identical, we combined the three studies to attain a sufficient sample size of Indigenous participants on the presupposition that both questions were a reasonable measure of experiences of racism.

As we were interested in exploring experiences of racism among the Indigenous survey participants, the primary independent variable of interest was Indigenous status. To determine Indigenous status, participants in all three surveys were asked “Are you of Aboriginal or Torres Strait Islander origin?” Participants who stated that they were Aboriginal (*n* = 328), Torres Strait Islander (*n* = 39) or both (*n* = 20) were combined.

The social determinants we investigated included socio-demographic characteristics and social capital. Socio-demographic variables included: age, sex, rurality, and three indicators of socioeconomic status (total annual household income, educational attainment, and employment status). Total annual household income included pre-tax income from all sources such as wages, social security payments, child support, and investments over the previous 12 months. Social capital included social support (ability to get help from family, friends and/or neighbours when needed), and social and civic trust.

Social trust was assessed by asking two questions: “Do you feel safe walking alone down your street after dark?”, and: “Do you agree that most people can be trusted?”. Civic trust was assessed by asking the following questions: “Do you feel valued by society?”, and: “Do you feel there are opportunities to have a real say on issues that are important to you?”

The lifestyle risk factors we investigated included smoking, alcohol consumption, unhealthy body weight, and physical inactivity. Survey participants disclosed their height and weight and their body mass index (BMI) was calculated. Underweight was defined as a BMI of less than 18.5 kg/m^2^, normal weight as a BMI of 18.5–24.9 kg/m^2^, overweight as a BMI of 25.0–29.9 kg/m^2^, and obesity as a BMI of 30 kg/m^2^ or more [11]. Physical inactivity was assessed using a series of questions and responses were compared against the National Physical Activity Guidelines for Australians to determine levels of physical inactivity [12].

### Missing data

Less than 5% of participants refused to answer or were unable to answer the survey questions for all variables; except for total annual household income (17%), body weight status (10%), physical activity level (7%), and feeling valued by society (6%). Missing data were included in all analyses as a separate category.

### Statistical analysis

We calculated weighted prevalence estimates for all variables with 95% confidence intervals (CI). We also calculated relative standard errors to determine the relative size of the sampling error and considered a relative standard error that exceeded 25% to be unreliable.

We used logistic regression to investigate the relationship between experiences of racism and Indigenous status. The dependent or outcome variable was experiences of racism (0 = never and 1 = at least once a year) and the primary independent or exposure variable of interest was Indigenous status (0 = no, 1 = yes and 9 = did not know or refused to say). We determined statistical significance at the *p* < 0.05 level.

We analysed the survey data with the Stata statistical software package version 12 [13], using the svy prefix commands that take into account the sampling design. We used the following steps:

1. Univariable logistic regression to identify independent variables that were associated with experiences of racism (Tables 1, 2 and 3).
2. Bivariable logistic regression to investigate the impact of each independent variable on the association between experiences of racism and Indigenous status (Table 4). We deemed that variables that increased or decreased the OR of the association between experiences of racism and Indigenous status by 10% or more were potentially explanatory variables [14].
3. Multivariable logistic regression (Table 4) to further investigate the contribution of all independent variables.

**Table 1:** Experiences of racism, by socio-demographic characteristics: univariable analysis

Variable	Experienced racism				Univariable analysis			
		% weighted prevalence			Crude odds ratio (OR)			
	n *	%	95% CI		OR	95% CI		*p* value
Indigenous status								
Non-Indigenous	1518	**4.5**	4.3	4.8	**1.0**	–	–	**–**
Indigenous	65	**17.0**	13.3	21.5	**4.3**	3.2	5.8	**<0.001**
Did not know or refused to say	13	**15.3**	8.9	25.1	**3.8**	2.0	7.1	**<0.001**
Indigenous status								
Anglo-Celtic **	576	**2.8**	2.6	3.0	**1.0**	–	–	**–**
Indigenous	65	**17.0**	13.3	21.5	**7.2**	5.3	9.7	**<0.001**
Did not know or refused to say	13	**11.7**	5.5	23.1	**4.6**	2.0	10.6	**<0.001**
Age (years)								
65+ years	294	**2.0**	1.8	2.3	**1.0**	–	–	**–**
55–64 years	363	**4.6**	4.2	5.2	**2.3**	2.0	2.8	**<0.001**
45–54 years	379	**6.8**	6.1	7.6	**3.5**	3.0	4.2	**<0.001**
35–44 years	333	**8.9**	8.0	10.0	**4.7**	4.0	5.6	**<0.001**
25–34 years	141	**10.5**	8.9	12.3	**5.6**	4.5	7.0	**<0.001**
18–24 years	86	**10.5**	8.5	12.9	**5.6**	4.3	7.3	**<0.001**
Sex								
Males	707	**5.3**	4.9	5.8	**1.0**	–	–	**–**
Females	889	**4.3**	4.0	4.6	**0.8**	0.7	0.9	**<0.001**
Geographic residence								
Metropolitan Victoria	874	**6.5**	6.1	6.9	**1.0**	–	–	**–**
Rural Victoria	722	**3.6**	3.3	3.8	**0.5**	0.5	0.6	**<0.001**
Total annual household income								
$100,000 or more	302	**5.3**	4.7	6.0	**1.0**	–	–	**–**
$40,000–$99,999	530	**5.1**	4.6	5.5	**1.0**	0.8	1.1	0.529
Less than $40,000	477	**4.0**	3.6	4.4	**0.7**	0.6	0.9	**<0.001**
Did not know or refused to say	287	**4.8**	4.2	5.4	**0.9**	0.7	1.1	0.200
Education								
Tertiary	637	**6.6**	6.1	7.1	**1.0**	–	–	**–**
Completed secondary	440	**5.0**	4.5	5.5	**0.7**	0.6	0.8	**<0.001**
Did not complete secondary	502	**3.3**	3.0	3.7	**0.5**	0.4	0.6	**<0.001**
Other/did not know/refused	17	**5.3**	3.3	8.6	**0.8**	0.5	1.3	0.392
Employment status								
Employed	905	**6.0**	5.6	6.4	**1.0**	–	–	**–**
Unemployed	95	**10.6**	8.6	13.1	**1.9**	1.5	2.4	**<0.001**
Not in the labour force ^#^	575	**3.2**	3.0	3.5	**0.5**	0.5	0.6	**<0.001**
Did not know or refused to say	21	**7.7**	4.9	12.0	**1.3**	0.8	2.1	0.276

*95% CI* = 95% confidence interval

*n = raw unweighted sample size; however, prevalence and odds ratio estimates are based on weighted data

**Comparison group is participants who were born in Australia to non-Indigenous Australian-born parents and only spoke English

# Included retirees (80%), home duties (10%), students (3%), and those who were unable to work (7%)

Bolding indicates that *p* value is significant at the *p* <0.05 level

**Table 2:** Experiences of racism, by social capital: univariable analysis

Measure of social capital	Experienced racism				Univariable analysis			
		% weighted prevalence			Crude odds ratio (OR)			
	n *	%	95% CI		OR	95% CI		*p* value
Able to get help from family?								
Yes	1026	**3.9**	3.7	4.2	**1.0**	–	–	**–**
Sometimes	274	**7.2**	6.4	8.2	**1.9**	1.6	2.2	**<0.001**
No	283	**9.4**	8.3	10.6	**2.5**	2.2	3.0	**<0.001**
Did not know or refused to say	13	**5.5**	3.1	9.5	**1.4**	0.8	2.6	0.255
Able to get help from friends?								
Yes	1086	**4.1**	3.8	4.3	**1.0**	–	–	**–**
Sometimes	342	**7.8**	7.0	8.7	**2.0**	1.7	2.3	**<0.001**
No	150	**7.1**	6.0	8.4	**1.8**	1.5	2.2	**<0.001**
Did not know or refused to say	18	**4.9**	3.0	7.9	**1.2**	0.7	2.0	0.485
Able to get help from neighbours?								
Yes	711	**3.5**	3.2	3.8	**1.0**	–	–	**–**
Sometimes	388	**6.6**	6.0	7.3	**2.0**	1.7	2.2	**<0.001**
No	452	**7.1**	6.4	7.8	**2.1**	1.8	2.4	**<0.001**
Did not know or refused to say	45	**5.2**	3.7	7.1	**1.5**	1.1	2.1	**0.020**
Believe most people can be trusted?								
Yes	446	**2.9**	2.6	3.2	**1.0**	–	–	**–**
Sometimes	696	**5.4**	5.0	5.8	**1.9**	1.7	2.2	**<0.001**
No	439	**9.6**	8.7	10.6	**3.6**	3.1	4.1	**<0.001**
Did not know or refused to say	15	**2.2**	1.3	3.8	**0.8**	0.4	1.3	0.341
Feel safe walking alone down street after dark?								
Yes	826	**4.1**	3.9	4.5	**1.0**	–	–	**–**
Sometimes	280	**7.0**	6.2	7.9	**1.7**	1.5	2.0	**<0.001**
No	411	**5.8**	5.2	6.4	**1.4**	1.2	1.6	**<0.001**
Not applicable	55	**2.5**	1.9	3.4	**0.6**	0.4	0.8	**0.001**
Did not know or refused to say	24	**4.7**	3.0	7.2	**1.1**	0.7	1.8	0.571
Feel valued by society?								
Yes	627	**3.5**	3.2	3.8	**1.0**	–	–	**–**
Sometimes	569	**6.0**	5.5	6.6	**1.8**	1.6	2.0	**<0.001**
No	321	**7.8**	7.0	8.8	**2.4**	2.0	2.7	**<0.001**
Did not know or refused to say	79	**3.9**	3.1	5.0	**1.1**	0.9	1.5	0.310
Feel there are opportunities to have a real say?								
Yes	414	**3.1**	2.8	3.4	**1.0**	–	–	**–**
Sometimes	573	**5.0**	4.6	5.4	**1.7**	1.4	1.9	**<0.001**
No	575	**7.6**	7.0	8.3	**2.6**	2.3	3.0	**<0.001**
Did not know or refused to say	34	**3.4**	2.4	4.8	**1.1**	0.8	1.6	0.591

*95% CI* = 95% confidence interval

*n = raw unweighted sample size; however, prevalence and odds ratio estimates are based on weighted data

Bolding indicates that *p* value is significant at the *p* <0.05 level

**Table 3:** Experiences of racism, by lifestyle risk factors: univariable analysis

Variable	Experienced racism				Univariable analysis			
		% weighted prevalence			Crude odds ratio (OR)			
	n *	%	95% CI		OR	95% CI		*p* value
Smoking status								
Non-smoker	838	**4.4**	4.1	4.7	**1.0**	–	–	**–**
Ex-smoker	455	**4.4**	4.0	4.9	**1.0**	0.9	1.1	0.964
Smoker	292	**7.1**	6.3	8.1	**1.7**	1.4	1.9	**<0.001**
Did not know or refused to say	11	**3.7**	2.0	7.0	**0.8**	0.4	1.6	0.617
Alcohol consumption								
Consumed alcohol in past 12 months	1166	**4.4**	4.1	4.7	**1.0**	–	–	**–**
Abstained from alcohol consumption	429	**5.8**	5.2	6.4	**1.3**	1.2	1.5	**<0.001**
Did not know or refused to say	1	**2.6**	0.4	16.1	**0.6**	0.1	4.2	0.581
Alcohol consumption (typical number of standard drinks per drinking session)								
1 or 2 standard drinks	749	**4.1**	3.8	4.4	**1.0**	–	–	**–**
3 or 4 standard drinks	258	**4.8**	4.2	5.4	**1.2**	1.0	1.4	0.060
More than 4 standard drinks	589	**5.7**	5.3	6.2	**1.4**	1.3	1.6	**<0.001**
Body weight status								
Normal weight	509	**4.3**	3.9	4.7	**1.0**	–	–	**–**
Underweight	38	**7.0**	5.0	9.7	**1.7**	1.1	2.4	**0.008**
Overweight	554	**4.8**	4.4	5.3	**1.1**	1.0	1.3	0.088
Obese	370	**5.0**	4.5	5.5	**1.2**	1.0	1.3	**0.048**
Did not know or refused to say	145	**4.6**	3.9	5.4	**1.1**	0.9	1.3	0.573
Physical activity								
Sufficient PA	609	**4.2**	3.9	4.6	**1.0**	–	–	**–**
Insufficient PA	832	**5.5**	5.1	5.9	**1.3**	1.2	1.5	**<0.001**
Sedentary	58	**3.2**	2.4	4.2	**0.8**	0.6	1.0	0.058
Did not know or refused to say	97	**3.9**	3.2	4.8	**0.9**	0.7	1.2	0.505

*95% CI* = 95% confidence interval

*n = raw unweighted sample size; however, prevalence and odds ratio estimates are based on weighted data

Bolding indicates that *p* value is significant at the *p* <0.05 level

**Table 4:** Impact of socio-demographic characteristics, lifestyle risk factors, and social capital on the association between perceived racism and Indigenous status; bivariable and multivariable analyses

Secondary independent variables	Adjusted Odds Ratio (OR)					
	Non-Indigenous	Indigenous				
		Adjusted OR	95% confidence interval		*p* value	% change from crude OR
Socio-demographic characteristics	1.0	3.5	2.5	4.8	**<0.001**	19%
Age	1.0	3.3	2.5	4.5	**<0.001**	23%
Sex	1.0	4.3	3.2	5.8	**<0.001**	0%
Geographic residence	1.0	4.5	3.3	6.0	**<0.001**	−3%
Socioeconomic status	1.0	4.5	3.3	6.0	**<0.001**	−3%
Total annual household income	1.0	4.4	3.3	5.9	**<0.001**	−2%
Highest level of education	1.0	4.8	3.6	6.5	**<0.001**	−11%
Employment status	1.0	4.1	3.0	5.5	**<0.001**	5%
Lifestyle risk factors	1.0	3.9	2.9	5.2	**<0.001**	10%
Smoking	1.0	3.9	2.9	5.3	**<0.001**	8%
Did not consume alcohol in past 12 months	1.0	4.2	3.2	5.7	**<0.001**	2%
Typical quantity of alcohol consumption	1.0	4.1	3.0	5.4	**<0.001**	6%
Obesity	1.0	4.2	3.2	5.7	**<0.001**	2%
Inadequate physical activity	1.0	4.4	3.3	6.0	**<0.001**	−3%
Social support	1.0	3.9	2.9	5.3	**<0.001**	9%
Inability to get help from family	1.0	4.0	3.0	5.3	**<0.001**	8%
Inability to get help from friends	1.0	4.1	3.1	5.6	**<0.001**	4%
Inability to get help from neighbours	1.0	4.1	3.1	5.5	**<0.001**	5%
Social and civic trust	1.0	3.7	2.7	5.0	**<0.001**	15%
Don’t believe most people can be trusted	1.0	3.8	2.8	5.1	**<0.001**	12%
Don’t feel safe walking alone down street after dark	1.0	4.4	3.3	5.9	**<0.001**	−2%
Don’t feel valued by society	1.0	4.1	3.0	5.5	**<0.001**	5%
Don’t feel there are opportunities to have real say	1.0	4.1	3.0	5.5	**<0.001**	6%
Multivariable model 1 ^a^	1.0	3.0	2.2	4.3	**<0.001**	29%
Multivariable model 2 ^b^	1.0	3.4	2.5	4.7	**<0.001**	21%
Multivariable model 3 ^c^	1.0	3.2	2.3	4.5	**<0.001**	26%

Dependent variable = experienced racism: crude odds ratio = 4.3 (95% CI: 3.1–5.8)

^a^Includes all secondary independent variables

^b^Includes secondary independent variables that changed the crude OR by 10% or more: age, education and one social trust indicator

^c^Includes the social determinants and excludes lifestyle risk factors

Bolding indicates that *p* value is significant at the *p* <0.05 level

## Results

Seventeen percent of Indigenous adults experienced at least one episode of racism in the year preceding the survey, compared with 4.5% of their non-Indigenous counterparts (Table 1). Thus, Indigenous adults living in Victoria were four times more likely than non-Indigenous adults to experience racism (odds ratio (OR) = 4.3; 95% CI = 3.2–5.8).

However, Victoria is a multicultural state with people from all over the world, including a large non-white non-Anglo-Celtic population who began to immigrate to Australia after the repeal of the White Australia Policy in 1973. By excluding participants who were not born in Australia to Australian-born parents and spoke a language other than English at home, we excluded the majority of the non-white non-Anglo-Celtic population who may similarly have experienced racism. While we assumed that there would be misclassification error, in the absence of any other data on ethnicity, the majority of those born in Australia to non-Indigenous Australian-born parents who only spoke English at home are likely at this point in time to be of the dominant white Anglo-Celtic population. This reduced the prevalence of experiences of racism in the non-Indigenous population from 4.5 to 2.8% (Table 1) and the OR of the association between experiences of racism and Indigenous status increased from 4.3 to 7.2 (5.3–9.7). Thus, Indigenous adults were 7 times more likely to experience racism than non-Indigenous adults who were born in Australia to Australian-born parents who only spoke English at home.

Socio-demographic variables that were significantly associated with experiences of racism included age, sex, rurality, and three indicators of socioeconomic status (Table 1). Age was inversely associated with experiences of racism; as age increased, experiences of racism decreased. Males were more likely than females to experience racism, as were those who lived in metropolitan compared with rural Victoria.

The association of experiences of racism with socioeconomic status varied according to the measure employed. Living in a household with a total annual income of less than $40,000, not being tertiary-educated, and not being in the labour force (unable to work, retired, engaged in home duties, or student) were associated with a lower prevalence of experiences of racism. In contrast, adults who were unemployed were almost twice as likely to experience racism as those who were employed.

Social support and trust are measures of ‘social capital’. There is no single definition of social capital. However, in essence social capital refers to the nature and extent of one’s social relationships across society, which determines access, or lack thereof, to the social and economic resources needed for a good life.

When we investigated the relationship of experiences of racism with social capital, we found that adults who were unable to get help when needed, irrespective of the source of help, were significantly more likely to experience racism than adults who were able to get help from any of these sources (Table 3). Similarly, social and civic trust were also associated with experiences of racism. Adults who did not believe that most people could be trusted were almost 4 times as likely as those that did believe most people could be trusted to experience racism; OR = 3.6 (3.1–4.1). Similarly, adults who did not feel safe walking alone down their street after dark, those who did not feel valued by society, and those who did not feel there were opportunities to have a real say on important matters, were more likely to experience racism.

When we investigated the relationship of experiences of racism and lifestyle risk factors, we found that adults who smoked, were underweight or obese, and who were physically inactive, were significantly more likely to experience racism than non-smokers, people of normal weight, and the physically active (Table 2). We used two measures of alcohol consumption and found a u-shaped relationship: Indigenous adults who abstained from alcohol consumption and those that drank excessively on any given occasion were both more likely to experience racism.

When we controlled for each secondary independent variable in a bivariable analysis; age, education, and social trust were the only three variables that changed the OR of the association between experiences of racism and Indigenous status by more than 10% and were thus deemed to be potentially explanatory of the association (Table 4).

In our study, twice as many non-Indigenous adults (44%) were aged 65 years and older compared with their Indigenous counterparts (22%). Controlling for age reduced the OR by 23% from 4.3 to 3.3 (2.5–4.5).

Similarly, a higher proportion of Indigenous adults (22%) than non-Indigenous adults (13%) did not believe that most people could be trusted. Controlling for social trust, reduced the OR by 12% from 4.3 to 3.8 (2.8–5.1).

In contrast, low educational attainment appeared to be protective against experiences of racism, as controlling for education increased the OR by 11% from 4.3 to 4.8 (3.6–6.5).

When we included all secondary independent variables in a multivariable analysis, the OR was reduced by 29% from 4.3 to 3.0 (2.2–4.3). However, the association between experiences of racism and Indigenous status remained highly significant at the *p* < 0.001 level (Table 4).

Controlling for the three variables deemed to be potentially explanatory (age, education, and social trust) in a multivariable model, reduced the OR by 21% from 4.3 to 3.4 (2.5–4.7).

Controlling for all social determinants reduced the OR by 26% from 4.3 to 3.2 (2.3–4.5). In contrast, controlling for all lifestyle risk factors only reduced the OR by 10% from 4.3 to 3.9 (2.9–5.2).

## Discussion

The prevalence of experiences of racism among Indigenous adults who lived in Victoria between 2011 and 2014, was 17% (13.3–21.5%), compared with 4.5% of non-Indigenous adults. Indigenous adults were four times more likely to experience racism than their non-Indigenous counterparts (OR = 4.3; 3.2–5.8). However, compared with the largely white non-Indigenous population of Anglo-Celtic origin, Indigenous adults were seven times more likely to experience racism (OR = 7.2; 5.3–9.7) as only 2.8% of Anglo-Celtic adults reported that they had experienced discrimination or been treated unfairly because of their racial, ethnic, cultural, or religious background. We expect that the estimate of 2.8% is likely to be an overestimate because some of these experiences of discrimination may have been due to religious background rather than race, ethnicity or culture.

Although we know that non-Indigenous adults of non-Anglo-Celtic origin also experience racism, our interest was specifically in the Indigenous experience of racism because of the enormous health inequities that exist between Indigenous and non-Indigenous adults. Whereas, the non-Indigenous non-Anglo-Celtic tend to be recent migrants who have better health than those born in Australia; commonly referred to as ‘the healthy immigrant effect’ [15].

To the best of our knowledge, we believe this study to be the first population-based study of experiences of racism among Indigenous adults who live in the state of Victoria.

However, we believe that our estimate of experiences of racism among Indigenous Victorians is a significant underestimate of the true prevalence of racism and that the estimate of 17% should be taken as meaning ‘at least’ 17%. We say this for the following reasons. Firstly, Indigenous status was determined by a simple single-item that asked, “Are you of Aboriginal or Torres Islander origin”. Some people may say yes to this question because they have a distant relative who was/is Indigenous, but they personally do not identify as Indigenous and may not ‘look’ Indigenous, given that there remains a widespread erroneous belief that indigeneity is about skin colour. Therefore, these individuals may not be at risk of experiencing racism due to their self-reported Indigenous origin. We have no way of distinguishing or quantifying such participants. If they made up a significant proportion of the Indigenous sample, then the prevalence of experiences of racism would be significantly underestimated. Indigeneity in Australia is generally determined by a three-part definition that must be met to be legally considered to be Indigenous. A person must have Indigenous heritage, identify as Indigenous and be accepted as such by an Indigenous community [16].

Secondly, the wider literature consistently shows that experiences of racism are typically under-reported [17]. Studies show that people are more likely to report experiences of racism if the question is phrased to ask about the experiences of the ethnoracial group to which they belong, rather than their personal experiences [17–19]. Evidence suggests this may be due to the psychologically protective effect associated with minimising personal experiences of racism [20, 21]. The Victorian Population Health Survey only inquired about a participant’s personal experiences of racism.

Thirdly, multi-item measures of experiences of racism tend to be more reliable than single-item measures [22]. For example, the 2014–15 National Aboriginal and Torres Strait Islander Social Survey, a population-based national survey that used a multi-item measure of experiences of racism, reported that 34% of Indigenous Australians experienced racism [23]. By contrast, the Victorian Population Health Survey only used a single-item measure.

It is important to be cognisant of the fact that racism is a complex phenomenon and reducing it to a single-item question cannot capture its complexity [24]. Indeed, its prevalence is highly likely to be underestimated when using a single-item question. Moreover, Indigenous people view racism as a more diverse and complex phenomenon than non-Indigenous people [25].

It is also well-known that survey questions developed for one culture may not be culturally appropriate for another culture. In recognition of this and the complexity of the phenomenon of racism, Paradies and Cunningham (2008) developed, tested and validated a 31-item Measure of Indigenous Racism Experiences (MIRE) to assess experiences of racism among Indigenous Australians [25]. Future research on the prevalence of experiences of racism among Indigenous Australians will likely be more accurate if it utilised the MIRE questions.

In 2011, the Localities Embracing and Accepting Diversity (LEAD) survey, conducted in Victoria, reported that 97% of Indigenous participants experienced racism [26]. The purpose of the LEAD study was not to specifically measure the prevalence of racism, but to investigate the relationship between experiences of racism and mental health outcomes. As a result, this study was not population representative of Victoria, as it was conducted in only four localities of Victoria and recruitment was non-random to maximise recruitment. However, as this study was conducted among specific Indigenous communities who met the three-part definition of indigeneity and almost all participants had experienced racism, its findings support our contention that our estimate is an underestimate of the true prevalence of racism experienced by Indigenous Victorians.

The time period of exposure to racism is also of importance. Our study asked about the previous 12 months while another study inquired about the lifetime prevalence of exposure to racism and estimated that 52.3% of Indigenous urban Victorians aged 12–17 years experienced racism [5].

Whether the prevalence of experiences of racism is higher or lower in Victoria compared with other states is currently unknown. However, we hypothesise that there may be a higher prevalence of experiences of racism in Victoria because Victoria has the lowest ethnic density (0.9%) of Indigenous Australians than any other state and there are only two discrete Indigenous communities in Victoria, which have small populations [27]. High own group ethnic density has consistently been shown to be protective against experiences of racism, believed to be due, at least in part, to a lower exposure to the perpetrators of racism [28–30].

Controlling for social determinants and lifestyle risk factors attenuated, but did not eliminate, the strong statistical association between experiences of racism and Indigenous status. Indigenous Victorians were still at least three times more likely to experience racism than their non-Indigenous counterparts after controlling for these factors.

The social determinants had a larger impact on the association between experiences of racism and Indigenous status than the lifestyle risk factors. The negligible impact of the lifetyle risk factors refutes the commonly made claim that the racism Indigenous Australians experience is due to their ‘poor lifestyle choices’, rather than their Indigenous status [7, 31]. This is particularly pertinent when considering alcohol consumption, as there is a commonly held negative stereotype that most Indigenous Australians drink alcohol to excess, often used to justify racism [31]. However, the evidence shows that Indigenous Australians are less likely to consume alcohol than non-Indigenous Australians and we found that Indigenous Victorians who abstained from alcohol consumption were as likely to experience racism as those who drank excessively [32].

Experiences of racism varied by socioeconomic status. Indigenous adults of low socioeconomic status, whether measured by household income, educational attainment or not being in the labour force, were less likely to experience racism than their higher socioeconomic counterparts. The single exception was that those who were unemployed were also more likely to experience racism. Our findings are consistent with the literature. For example, the Darwin Region Urban Indigenous Diabetes (DRUID) study also found a higher prevalence of experiences of racism among Indigenous Australians of high socioeconomic status [33].

A possible explanation of why experiences of racism are higher among Indigenous Australians of higher socioeconomic status may be that those who manage to overcome the substantial barriers that Indigenous people continue to face in mainstream society are a minority within a minority. This is likely to increase an individual’s exposure to mainstream society and put them at a greater likelihood of experiencing racism, which is consistent with the evidence on the protective effects of high own group ethnic density. It may also explain the seeming contradiction of unemployed Indigenous Victorians being more likely to experience racism. Unemployed Indigenous Victorians may also have a higher exposure to mainstream society because such exposure is necessary to receive unemployment benefits. Alternatively, or additionally, it is possible that people of higher socioeconomic status have a greater propensity to report experiences of racism.

Our findings that low educational attainment appear to be protective against experiences of racism is of concern given the poorer socioeconomic outcomes associated with low levels of education. There is a large body of research demonstrating the existence of maladaptive problem-focused behavioural responses to racism, such as opting out of formal education as an act of self-protection [34]. This may help to explain the lower secondary school completion rates among Indigenous children and is supported by a recent study in Victoria, which identified racism within the school system as one of the most challenging issues faced by Indigenous children, particularly at secondary school level [35].

The implication of this finding is that more needs to be done to eradicate systemic and interpersonal racism within our education system. In 2012, the Race Discrimination Commissioner observed that: “Sometimes racism can be reflected in not telling the history of an event or the experience of a group of people in our country” [36]. Currently, what is, or is not, taught in schools about Indigenous history and culture, depends on individual schools. Unfortunately, an attempt to introduce a national curriculum, which embedded education about Indigenous culture, history, and the impact of colonisation, was thwarted in 2014 by the Federal government, following a non-independent review of its content [37].

At a societal level, groups who claim ethnoracial superiority at the expense of those deemed inferior, derive great benefits from the inequitable social and economic living conditions that are generated [17]. However, for the group deemed to be inferior, chronic experiences of racism are harmful to their mental and physical health [38–40]. While racism is not always intentional and much of systemic racism is carried out by people who are ignorant or in denial, this doesn’t lessen its harmful effects.

According to Krieger’s ecosocial analysis, the harm occurs through seven pathways [17]: (1) economic and social deprivation; (2) higher exposure to toxins, hazards, and pathogens; (3) social trauma, (4) health-harming responses to racism, (5) targeted marketing of harmful products; (6) inferior and inadequate health care; and (7) environmental degradation and alienation from the land [22].

Strengths of our study include that it was based on data from the Victorian Population Health Survey, which has been conducted annually since 2001 and is a well-validated population-based survey with a relatively high response rate. Moreover, the Victorian Population Health Survey collects data on a wide breadth of topics, including the social determinants of health, because it was informed by a public health model of the social determinants of health [41]. In contrast, most health surveys tend to be informed by the biomedical model of health, which attributes disease to proximate biological factors at the level of the individual and ignores the social determinants of health. Collecting data on the social determinants of health provdes an opportunity to develop policy directions that address racism.

Weaknesses of the study, other than those previously described, include the use of two different questions about experiences of racism. While the questions in the 2011 and 2012 studies ask specifically about experiences of racism directly attributable to Indigenous status, the question in the 2014 survey asked about experiences of racism attributable to ‘racial, ethnic, cultural, or religious background’. This leaves open the question of potential intersectionality between race and religion and whether we are accurately measuring experiences of racism among participants from the 2014 survey which would impact on the prevalence estimate of the combined dataset. The prevalence of experiences of racism among Indigenous participants from the 2011 and 2012 surveys was 19.6% (13.8–27.1%) compared with 15.0% (10.7–20.7%) from the 2014 survey. Although lower among the 2014 Indigenous participants, the difference was not statistically significant.

Survey data is cross-sectional, which does not allow for conclcusions to be drawn in relation to cause and effect or its direction. For example, feeling unsafe walking alone after dark could be the consequence and/or cause of self-reported racism.

The data is self-reported raising concerns about bias and accuracy. However, not all data readily lends itself to objective measurement, and experiences of racism are an example [34]. However, it is self-reported racism that is strongly associated with mental and physical ill-health [42].

The Victorian Population Health Surveys conducted prior to 2015 only surveyed households with landline telephone connections. Yet the exponential uptake of mobile telephones has caused a rapid growth in households that rely solely on mobile telephones and raised concerns that telephone surveys that only include fixed landline connections are losing their population representativeness [43]. Moreover, Indigenous women have been found to be five times more likely than non-Indigenous women to live in mobile-only households [43]. Therefore, if the experiences of Indigenous households who have landline telephones are different to those who do not, our findings may not be as population representative as we suppose.

As noted by the extensive work of Maggie Walter, the collection, analysis and interpretation of data on Indigenous peoples are not as objective as non-Indigenous peoples claim them to be [44]. In Australia most research is conceived, conducted and interpreted by non-Indigenous people who are largely of middle class Ango-Celtic origin. Consequently, the research decisions made reflect the social norms, values and beliefs of the non-Indigenous. This has lead to a lot of research that effectively stigmatises Indigenous people, thus reinforcing racism.

For example, there is a disproportionate amount of research that focuses on health behaviours such as smoking and alcohol consumption, comparing Indigenous with non-Indigenous people. Such research concludes that Indigenous people are more likely to engage in unhealthy health behaviours than their non-Indigenous counterparts, which is stigmatising [45]. This has lead to policies aimed at closing the gap in health between Indigenous and non-Indigenous Australians being almost exclusively focused on reducing the gap in health behaviours, which is notoriously hard to do in any population. Yet the irony of this is that health behaviours only account for approximately 32% of the total burden of disease and this itself may be an overestimate as it is based on a study that only included health behaviours in the risk factors analysis [46]. It is the social determinants of health that account for a far greater proportion of the burden of disease.

The reasons for this not only reflect the dominance of the biomedical model of health, which was conceived in Europe and the United States, but also Western neoliberal culture that values individualism over collectivism and regards individual responsibility as the pathway to good health. It is at odds with the Indigenous perspective on health. Imposing such beliefs and values through prioritising this type of research is, arguably, racist. We therefore recognise this as a weakness of our study and join the growing call for better engagement with, and the inclusion of, Indigenous people and Indigenous researchers at all stages of the research process, from conception to publication.

Each year the Prime Minster of Australia reports on progress towards closing the gap in Indigenous health. However, in the 9 years since the commencement of the ‘Closing the Gap’ strategy, very little has been achieved and in some cases the gap is widening [47]. The National Indigenous and Torres Strait Islander Health Plan 2013–2023, designed to address the gap, acknowledges that “racism is a key social determinant of health for Indigenous and Torres Strait Islander people…” and seeks to eliminate systemic racism within the healthcare sector. However, it still disproportionately focuses on changing the health behaviours of Indigenous Australians and ignores the wider systemic and interpersonal racism directed against Indigenous Australians [48]. We contend that the gap is unlikely to be reduced until we comprehensively address racism towards Indigenous Australians [49].

A large body of work on anti-racism strategies and interventions has been conducted and trialled by the Victorian Health Promotion Foundation [50]. We refer readers to their website [50]. Table 5 attempts to summarise potential policies and interventions, by sector, that may effect real societal change in attitudes and behaviours. The list is not meant to be exhaustive, but rather to provoke thought. Many of the policies and interventions are aimed at eliminating systemic racism rather than interpersonal racism which is the subject of this study. However, all forms of racism ought to be tackled simultaneously to prevent reversion. Moreover, piecemeal approaches to tackling racism that are often underfunded and not sustained have the potential to do more harm than good [51].

**Table 5:** Potential policies and interventions to eradicate racism

Sector	Policies/interventions
Health	Mandatory anti-racism and cultural competency training for all staff in mainstream health services with periodic refresher courses.
	A surveillance system to monitor treatment in mainstream services by Indigenous status, linked to health outcomes and reported to an independent committee.
	Expansion of culturally safe Indigenous community controlled health organisations.
Police, courts, and corrective services	Mandatory anti-racism and cultural competency training for all staff with periodic refresher courses.
	Full implementation of the 339 recommendations of the 1987 Royal Commission into Aboriginal deaths in custody.
	Properly fund good quality legal services, reinstate and expand Indigenous language translation services.
	Policies of non-tolerance of racial discrimination with consequences.
	Increase opportunities for, and active recruitment of Indigenous police officers.
	Legislation to ban the incarceration of Indigenous people for unpaid fines and other minor offences.
	Surveillance system to compare Indigenous and non-Indigenous incarceration rates by crime that is routinely reported to an independent committee.
Media	Anti-racism social marketing campaigns.
	Social marketing campaigns to encourage bystander action in racist incidents.
	Mandatory anti-racism and cultural competency training for all media staff with periodic refresher courses.
Education	Mandatory anti-racism and cultural competency training for all staff, with periodic refresher courses.
	Implement a national curriculum that provides for the mandatory teaching of Indigenous culture, history, the impact of colonisation, and the impacts of racism.
	Increase opportunities for, and active recruitment of Indigenous teachers.
Government and public service	Reinstate the Australian and Torres Strait Islander Commission (ATSIC) or its equivalent and implement the recommendations of the Uluru Statement from the heart.
	Recognise and openly acknowledge that racism is endemic within our institutions and society, and is a significant health risk factor for Indigenous people.
	Adopt a human rights-based approach to policy-making for Indigenous Australians.
	Mandatory anti-racism and cultural competency training for all staff and members of parliament with periodic refresher courses.
	Properly fund the native title system.
	Correct reporting of government expenditure on Indigenous-specific services with an active media campaign to dispel the myth of the ‘wasted millions’.
Housing	Replace degraded housing stock and provide additional housing to reduce overcrowding.
Institutions	Implement place-based anti-racism interventions such as the Localities Embracing and Accepting Diversity (LEAD) intervention (VicHealth 2014).

In Australia, a paternalistic ideology continues to pervade policy-making for Indigenous Australian across all levels of government [52]. This is, therefore a key area for reform. Paternalistic policies are inherently racist as they do not recognise the right to self-determination and seek to limit the choices of individuals, based on the belief that individuals do not know what is in their best interest. The antithesis of the paternalistic approach is a human rights-based approach. The adoption of a human rights-based approach to policy-making would be more likely to facilitate the elimination of systemic racism which in turn would lead to better health and wellbeing outcomes for Indigenous people.

## Conclusions

This study shows that, contrary to the current discourse in Australia that denies the existence of racism, racism directed against Indigenous adults in Victoria is a significant problem and may be associated with lower educational attainment, which may lead to lower socioeconomic status and poorer health outcomes.

Therefore, if as a society we truly wish to reduce the gap in health between Indigenous and non-Indigenous Australians, we should: (a) acknowledge that racism against our Indigenous counterparts exists; (b) that it is extensive and harmful; and (c) that it is a major determinant of the gap in health. Moreover, racism directed against Indigenous Australians is a problem that needs to be addressed by the dominant non-Indigenous population through challenging and changing beliefs and behaviours in schools, workplaces, the media, the public sector, government and society at large.

## Abbreviations

ABS: Australian Bureau of Statistics
BMI: body mass index
LEAD: The Localities Embracing and Accepting Diversity survey
VicHealth: Victorian Health Promotion Foundation
